# Toxicity Assessment of New Ag-ZnO/AgO Nanocomposites: An In Vitro and In Vivo Approach

**DOI:** 10.3390/jfb15030051

**Published:** 2024-02-20

**Authors:** José Rodrigues do Carmo Neto, Pablo Igor Ribeiro Franco, Yarlla Loyane Lira Braga, Jordana Fernandes de Oliveira, Hugo Felix Perini, Luís Fernando Duarte Albuquerque, Danieli Brolo Martins, Fernanda Rodrigues Helmo, Anderson Assunção Andrade, Marina Pacheco Miguel, Mara Rúbia Nunes Celes, Thiago Lopes Rocha, Anielle Christine Almeida Silva, Juliana Reis Machado, Marcos Vinícius da Silva

**Affiliations:** 1 Department of Bioscience and Technology, Institute of Tropical Pathology and Public Health, Federal University of Goias, Goiânia 74605-050, GO, Brazil; rodriguesnneto@gmail.com (J.R.d.C.N.); pablo_igor@hotmail.com (P.I.R.F.); yarlla_lira@hotmail.com (Y.L.L.B.); jordana.fer@hotmail.com (J.F.d.O.); mrubia_celes@ufg.br (M.R.N.C.); juliana.patologiageral@gmail.com (J.R.M.); 2 Department of Microbiology, Immunology and Parasitology, Institute of Biological and Natural Sciences of Federal University of Triângulo Mineiro, Uberaba 38025-180, MG, Brazil; pos-doc.hugoperini@uftm.edu.br (H.F.P.); anderson.andrade@uftm.edu.br (A.A.A.); 3 Programa de Pós-Graduação em Ciências Animal, School of Veterinary and Animal Science, Federal University of Goias, Goiânia 74690-900, GO, Brazil; luis_albuquerque@discente.ufg.br (L.F.D.A.); danieli@ufg.br (D.B.M.); marinapacheco@ufg.br (M.P.M.); 4 Department of General Pathology, Federal University of Triângulo Mineiro, Uberaba 38025-180, MG, Brazil; fernandahelmo@gmail.com; 5 Laboratory of Environmental Biotechnology and Ecotoxicology, Institute of Tropical Pathology and Public Health, Federal University of Goiás, Goiânia 74605-050, GO, Brazil; thiagorochabio20@gmail.com; 6 Laboratório de Novos Materiais Nanoestruturados e Funcionais (LNMIS), Physics Institute, Federal University of Alagoas, Maceió 57072-900, AL, Brazil; aniellechristineas@gmail.com

**Keywords:** nanoparticles, subacute toxicity test, in vitro techniques, nanocomposites, zinc oxide (ZnO), silver (Ag)

## Abstract

Zinc oxide nanoparticles (ZnO NPs) are metal oxide nanomaterials, which are important for several applications: antibacterial, anthelmintic, antiprotozoal and antitumoral, among others. These applications are mainly related to the ability to spontaneously produce and induce the production of reactive oxygen species that are important components for the destruction of pathogens and tumor cells. While trying to potentiate ZnO NPs, studies have associated these NPs with silver oxide (AgO) or silver (Ag) NPs. It has already been reported that this combination (Ag-ZnO/AgO NPs) is able to enhance the microbicidal potential. Although possessing much potential for several purposes, it is important to evaluate whether this association also poses the risk of toxicity to cells and experimental models. Therefore, this work aimed to evaluate the toxicity of various Ag-ZnO/AgO NP nanocomposites, in vitro and in vivo. Accordingly, ZnO nanocrystals and nanocomposites with various concentrations of AgO (ZnO:5Ag, ZnO:9Ag or ZnO:11Ag) were used in different cytotoxicity models: *Galleria mellonella* (*G. mellonella*), cell lines (VERO and RAW 264.7) and C57BL/6 mice. In the *G. mellonella* model, four concentrations were used in a single dose, with subsequent evaluation of mortality. In the case of cells, serial concentrations starting at 125 µg/mL were used, with subsequent cytotoxicity assessment. Based on the safe doses obtained in *G. mellonella* and cell models, the best doses were used in mice, with subsequent evaluations of weight, biochemistry as also renal and liver histopathology. It was observed that the toxicity, although low, of the nanocomposites was dependent upon the concentration of AgO used in association with ZnO NPs, both in vitro and in vivo.

## 1. Introduction

Nanoparticles (NPs) are nanoscale materials, which accommodate the dynamic electrical, magnetic, morphological and chemical characteristics of any substance [1]. Due to their reduced size, they exhibit a larger surface area, consequently yielding a higher number of free atoms and enhancing the reactivity of substances [1]. This can lead to direct cytotoxic effects on microorganisms [2].

The U.S. Food and Drug Administration (FDA) has deemed bulk zinc oxide (ZnO) to be a safe and non-toxic substance [3]. Zinc oxide nanoparticles (ZnO NPs) have been used for some time across various formulations, such as sunscreens, foot-care products and ointments, in numerous topical products, pigments and coatings and even as a fungicide in paints [4]. Thus, the use of this type of material is widespread in the community and in the health sector.

Metal oxide NPs, such as ZnO, induce the production of reactive oxygen species [5], which are important for anthelmintic and antiprotozoal activities, as seen in example [6]. In an attempt to potentiate the varied actions of ZnO NPs, studies have associated these NPs with silver (Ag) or silver oxide (AgO) NPs [7,8]. It has already been reported that this combination (Ag-ZnO/AgO NPs) was able to enhance the microbicidal potential against *Prevotella intermedia*, *Salmonella* sp. [9] and *Leishmania braziliensis* [10]. Further, Ag-ZnO/AgO NPs have been shown to have antioxidant, anti-inflammatory and skin-lesion healing potential when administered topically in mice [11]. While showcasing considerable potential for various applications, it is imperative to assess whether this amalgamation poses any risks or toxicity to healthy cells, both for in vitro and experimental models. Consequently, the primary aim of this study was to comprehensively evaluate the toxicity of various Ag-ZnO/AgO NP nanocomposites, while examining their impact in vitro on cell lines and in vivo through subacute toxicity models.

## 2. Materials and Methods

### 2.1. Nanomaterials

Pure ZnO nanocrystals and Ag-ZnO/AgO nanocomposites were prepared using the method under patent application (BR 10 2018 0077147). The nanocomposites used here were named ZnO:5Ag (49%), ZnO:9Ag (65%) and ZnO:11Ag (68%); this nomenclature corresponds to the percentage of AgO nanocrystals found in their structure. Structural and morphological properties of ZnO nanocrystals and Ag-ZnO/AgO nanocomposites were previously characterized by [9,10] and are shown in Table 1.

### 2.2. Evaluation of Nanomaterial Toxicity in a Galleria mellonella (G. mellonella) Model

To evaluate the impact of nanomaterials on an alternative non-mammalian model, last instar *G. mellonella* larvae weighing between 250 mg and 300 mg, displaying no color alteration or change in motility, 10 larvae per group, were treated using nanoformulations. The region of the last proleg was cleaned using 70% ethanol before inoculating the treatments and nanocomposites (ZnO, ZnO:5Ag, ZnO:9Ag and ZnO:11Ag) diluted in phosphate-buffered saline (PBS vehicle) at a final concentration of 5, 10, 50 and 100 mg/kg per larva in a single dose. Larvae from the control group received only PBS (vehicle). Larvae were maintained at 37 °C in the absence of light, and their survival was monitored for 8 days at 24 h intervals. The experiments were conducted in triplicate.

### 2.3. Cytotoxicity Evaluation of Nanomaterials in VERO and RAW 264.7 Cell Lines

VERO and RAW 264.7 cells were maintained in a complete Roswell Park Memorial Institute (RPMI) medium (with 10% FBS and 40 μg/mL gentamicin) at 37 °C and 5% carbon dioxide (CO_2_). The cells come from the cell bank of the Laboratório de Pesquisa em Parasitologia e Biologia Molecular/DMIP/UFTM. The maintenance of the cells was performed as needed, starting from the establishment of a confluence above 80%. Before biological testing, the nanomaterials were solvated in dimethyl sulfoxide (DMSO) with a mortar and agate pestle. Subsequently, they were diluted in ultrapure water until each compound reached a concentration of 3.33 mg/mL (standard aliquot dose that was subsequently diluted according to each experiment).

VERO or RAW 264.7 cells were plated in 96-well culture plates at a concentration of 5 × 10^5^ cells/well. Post cell adhesion, the supernatant was discarded to remove nonadherent cells, which were exposed to treatments using ZnO, ZnO:5Ag, ZnO:9Ag or ZnO:11Ag in serial concentrations (125 to 0 µg/mL). Following 24 h of treatment, 2.5 mg/mL of resazurin (C_12_H_7_NO_4_) was added per well, and fluorescence was evaluated in an EnSpire^®^ reader (Perkin Elmer, Rodgau, Germany) at 550–590 nm to determine the toxic concentration capable of killing 50% of the cells (CC50). In addition to this parameter, the selectivity index was also defined by dividing the CC50 of RAW cells by the CC50 of VERO cells to assess selectivity via immune and nonimmune cells.

### 2.4. Toxicity Evaluation of Nanomaterials in C57Bl/6 Mice

#### 2.4.1. Animals

C57Bl/6 mice, 8 to 10 weeks old, weighing 22–27 g each, were used for the experiment. The animals were kept in the Centro Multiusuário de Produção e Experimentação Animal (CMPEA/IPTSP/UFG) under controlled and known conditions: in polypropylene cages of 414 mm × 168 mm, at a temperature between 20 °C and 25 °C, a humidity between 45% and 55%, and with constant air renewal. They were fed with a feed of known composition (Nuvilab-CR1, NUVITAL, Paraná, Brazil), and water was offered ad libitum. All cage components, such as water and wood shavings for foraging and feed, were autoclaved before use and exposure to the animals. The work was submitted and approved (Protocol No. 091/20; date: 03/22/2021) by the Comissão de Ética no Uso de Animais (CEUA) da Universidade Federal de Goiás (UFG), and all ethical aspects of animal handling were followed. In addition, the research group involved in the work was attentive to the behavior of the animals, on a daily basis, for the identification of humane endpoints if necessary.

#### 2.4.2. Groups and Treatment Regimen

For subacute toxicity evaluation, animals were randomly divided into 14 groups (n = 4).

The following groups were maintained for 8 days:

1: Control group administered sterile PBS

2: Group treated with ZnO (5 mg/kg/d)

3: Group treated with ZnO:5Ag (5 mg/kg/d)

4: Group treated with ZnO:9Ag (5 mg/kg/d)

5: Group treated with ZnO:11Ag (5 mg/kg/d)

6: Group treated with ZnO (10 mg/kg/d)

7: Group treated with ZnO:5Ag (10 mg/kg/d) 

8: Group treated with ZnO:9Ag (10 mg/kg/d)

9: Group treated with ZnO:11Ag (10 mg/kg/d)

The following groups were maintained for 20 days:

10: Control group administered sterile PBS

11: Group treated with ZnO (5 mg/kg/d)

12: Group treated with ZnO:5Ag (5 mg/kg/d)

13: Group treated with ZnO:9Ag (5 mg/kg/d)

14: Group treated with ZnO:11Ag (5 mg/kg/d)

Before treatment, the NPs were dispersed in sterile PBS and subjected to vortex agitation for 3 min (300 rpm). Nanomaterials were administered by gavage (100 µL/animal) for seven consecutive days at 24 h intervals. Weight was monitored before and 8 or 20 days after the beginning of the treatment. Euthanasia was performed through cervical dislocation (after sedation with 5% xylazine hydrochloride and 10% ketamine solution intraperitoneally). Subsequently, blood was collected via venipuncture with subsequent transfer of serum to a 2 mL tube and stored at −80 °C for biochemical analysis. During necropsy, the viscera were evaluated macroscopically, whereas the livers and kidneys were collected for histological analysis.

#### 2.4.3. Histological Analyses

The liver and kidney of the specimens were fixed in 4% buffered paraformaldehyde for a maximum of 48 h. They were then transferred to an ascending series of ethyl alcohols for dehydration, followed by clearing in xylene and embedding in paraffin. The fragments were positioned in a longitudinal axis perpendicular to the microtomy plane, to obtain transverse sections. Serial cuts 5 μm thick, with each cut 25 μm apart from the other (n = 3), were made; these were fixed to the slides using polylysine adhesive and subjected to drying. The material was then subjected to routine hematoxylin–eosin staining. 

Using a common light microscope coupled to a camera, five photos of each serial fragment were captured under 400× magnification for subsequent analysis (n = 15/animal). For each organ, a series of parameters were used to define the histopathological alteration index (HAI). In the HAI, the changes were classified into progressive stages corresponding to the impairment of the organ’s functions: stage I changes, which did not compromise the functioning of the organ; stage II, more severe and which impaired the normal functioning of the organ, but were reversible; and stage III, very severe and irreversible changes, adapted from [12,13]. An HAI value was calculated for each animal using the formula: HAI = (1 × ΣI) + (10 × ΣII) + (100 × ΣIII), with I, II and III corresponding to the number of stage I, II and III changes, respectively. The average HAI value was divided into 5 categories: 0–10 = normal tissue function; 11–20 = mild-to-moderate tissue damage; 21–50 = moderate-to-severe tissue modification; 51–100 = severe tissue modification; greater than 100 = irreparable damage to the tissue. This index allowed a comparison of the severity of organ injuries. The analyses were carried out independently and blindly by two evaluators.

#### 2.4.4. Evaluations of Biochemical Parameters

Analyses of serum biochemical parameters were performed using automation methodology via an apparatus (CM 250 automatic analyzer —Wiener lab^®^, Rosário, Argentina), following the manufacturer’s instructions (Roche Diagnostics Ltd., Rotkreuz, Switzerland). Thus, the levels of aspartate transaminase (AST) (mg/dL) (BioTécnica, Varginha, Brazil, Lot. 13S15B), alanine transaminase (ALT) (mg/dL) (BioTécnica, Lot. 16N16B) and creatinine (mg/dL) (Doles, Goiânia, Brazil, Lot. CRTB 21111) were quantified in the animals’ serum.

### 2.5. Statistical Analysis

The database was created using the EXCEL 2016 software, whereas the statistical analyses were performed using GraphPad Prism 8.0.1 (Prism 8.0.1, GraphPad Software, San Diego, CA, USA). The verification of the normal distribution of quantitative variables was assessed using the Shapiro-Wilk test. For comparisons between the two groups, the unpaired “t” test was used for data with a normal distribution, and the Mann-Whitney test was used for data with a non-normal distribution. For comparisons among more than two groups, the two-way ANOVA test was used for data with a normal distribution, and the Tukey test was used for data with a non-normal distribution. Survival curve construction was performed using the Kaplan-Meier analysis, whereas significant differences were determined using the log-rank test. The results were considered statistically significant at *p* < 0.05.

## 3. Results

### 3.1. Ag content Increases the Cytotoxicity of Nanocomposites in an Invertebrate Model

**Figure 1:**
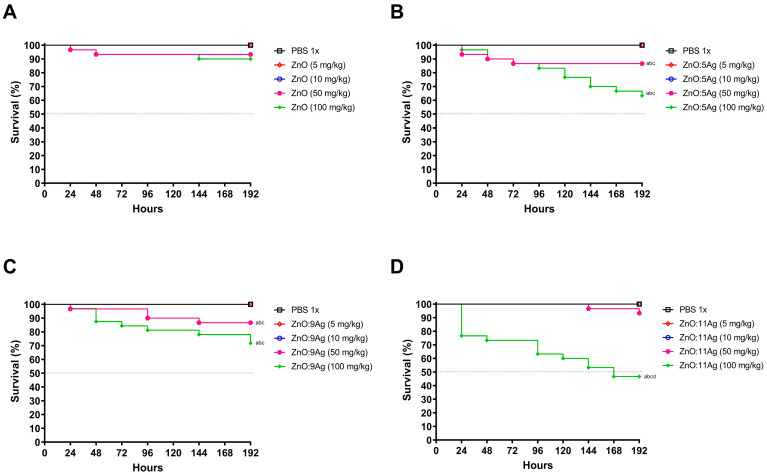
Impact of treatment with ZnO nanocrystals (**A**), or nanocomposites of ZnO:5Ag (**B**), ZnO:9Ag (**C**) or ZnO:11Ag (**D**) on the survival of *Galleria mellonella* (nm = 30/concentration). Treatments were administered via the left last proleg, in a single dose, at concentrations of 5 mg/kg, 10 mg/kg, 50 mg/kg or 100 mg/kg, and survival was followed for 192 h/8 days. Survival curve construction was performed using Kaplan-Meier analysis, and significant differences were determined using the log-rank test. Differences were considered statistically significant at *p* < 0.05: (a) difference between treatments and vehicle of PBS; (b) difference between treatment and concentration of 5 mg/kg; (c) difference between treatment and concentration of 10 mg/kg vehicle; and (d) difference between treatment and concentration of 50 mg/kg.

To evaluate the toxicity of nanomaterials in a non-mammalian multicellular model, *G. mellonella* larvae were exposed to four different concentrations at a single dose, and their survival was monitored for 8 days (Figure 1). In the case of all nanomaterials, only concentrations of 50 mg/kg and 100 mg/kg were able to induce mortality in the larvae, both compared to the vehicle-treated group and the concentrations of 5 mg/kg and 10 mg/kg. However, concentrations of 5 mg/kg and 10 mg/kg showed no impact on invertebrate survival.

### 3.2. Ag Content Increases the Cytotoxicity of Nanocomposites in RAW 264.7 and VERO Cell Lines

To evaluate the impact of nanomaterials and define the CC50, two different cell lines (RAW 264.7 or VERO) were exposed to the treatments for 24 h (Table 2). Accordingly, it was noted that all nanomaterials demonstrated a concentration-dependent profile. Further, the ZnO:9Ag nanocomposite proved to be the most toxic to both cell lines, with a CC50 of 28.40 μg/mL for RAW 264.7 cells and 26.16 μg/mL for VERO cells (Table 2). The ZnO:11Ag nanocomposite was found to be the second most toxic, with a CC50 of 58.84 μg/mL for RAW 264.7 cells and 47.39 μg/mL for VERO cells, indicating that a higher Ag concentration resulted in a more cytotoxic profile when associated with ZnO in vitro (Table 2). The ZnO nanocrystal and ZnO:5Ag nanocomposite demonstrated similar CC50 values for both cell lines, with a range of 95.59 to 120 μg/mL. In addition, the selectivity index indicates a low selection and differentiation of treatments between immune (RAW 267.4) and nonimmune (VERO) cells, with a higher Ag concentration associated with a higher selectivity index (Table 2).

### 3.3. Impact of Treatments during the Subacute Phase in C57Bl/6 Mice

Since doses of 5 mg/kg and 10 mg/kg did not demonstrate an impact on *G. mellonella* survival, these two doses were selected for further evaluation in a murine model. In addition, it was evaluated whether the pattern of toxicity observed for the ZnO:9Ag and ZnO:11Ag nanocomposites both in vitro and in *G. mellonella* would be maintained in mice.

**Figure 2:**
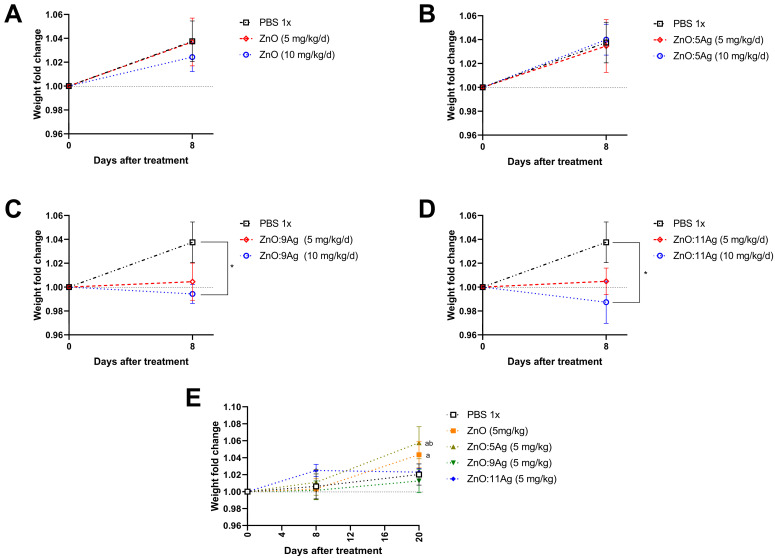
Impact of treatment with ZnO nanocrystals (**A**), or nanocomposites of ZnO:5Ag (**B**), ZnO:9Ag (**C**) or ZnO:11Ag (**D**) on weight gain of male C57Bl/6 mice (n = 4). Treatments were administered via gavage for seven consecutive days at doses of 5 mg/kg/d or 10 mg/kg/d, and weight was collected on the first day before treatment, after 8 days (**A**,**B**) or after 20 days (**E**). A two-way ANOVA test (Sidak’s multiple comparisons test) was performed. Differences were considered statistically significant when *p* < 0.05. * indicates a difference between treatments and vehicles; (a) the difference between 20 days and 0 days; (b) the difference between 8 days and 20 days.

Accordingly, C57Bl/6 mice were treated using ZnO nanocrystals or nanocomposites of ZnO:5Ag, ZnO:9Ag or ZnO:11Ag for seven consecutive days at two doses: 5 mg/kg/d or 10 mg/kg/d and euthanized 8 days after the beginning of treatment. Both ZnO nanocrystals (5 mg/kg/d, *p* = 0.9993; 10 mg/kg/d, *p* = 0.7115) (Figure 2A) and the ZnO:5Ag nanocomposite (5 mg/kg/d, *p* = 0.9847; 10 mg/kg/d, *p* = 0.9957) (Figure 2B) did not alter the weight gain of the animals. However, following the same pattern observed in vitro and in the invertebrate model, exposure to 10 mg/kg/d of the nanocomposites ZnO:9Ag (*p* = 0.0172) (Figure 2C) and ZnO:11Ag (*p* = 0.0140) (Figure 2D) was able to significantly reduce weight gain compared to the vehicle-treated group after 8 days. However, the dose of 5 mg/kg/d was not able to significantly reduce the weight of animals (ZnO:9Ag, *p* = 0.0754; ZnO:11Ag, *p* = 0.1426), demonstrating a dose-dependent pattern of these two types of nanomaterials.

Since the 10 mg/kg dose demonstrated an impact on animal weight for the ZnO:9Ag and ZnO:11Ag nanocomposites, the 5 mg/kg dose was selected to evaluate the impact of these nanomaterials for a longer period after treatment. In this case, C57Bl/6 mice were treated for seven consecutive days with each nanomaterial and euthanized 20 days after the start of treatment (Figure 2E). Regarding weight, regardless of the type of nanomaterial and AgO concentration, no group had weight gain impairment. Interestingly, mice treated with ZnO exhibited weight gain after 20 days of observation (*p* = 0.0441) compared to the first day of treatment. The same pattern was observed for the ZnO:5Ag-treated group, wherein mice gained weight after the same period, both when compared to the first day of treatment (*p* = 0.0055) and 8 days after the start of the intervention (*p* = 0.0288) (Figure 2E).

**Figure 3:**
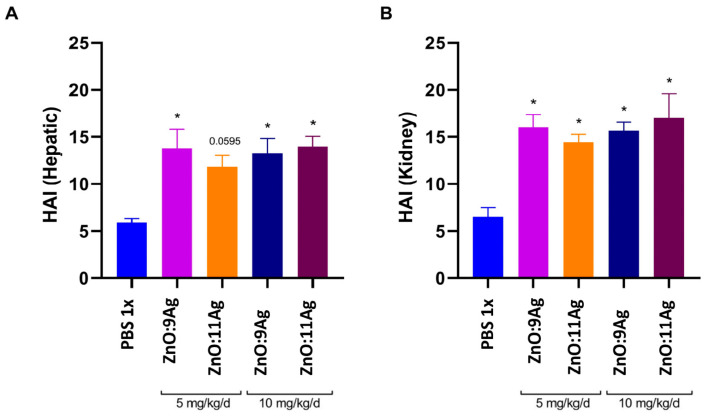
HAI of liver (**A**) and kidney (**B**) histopathological changes in male C57Bl/6 mice (n = 4) after treatment with zinc oxide nanocrystals and ZnO:9Ag or ZnO:11Ag nanocomposites. Treatments were administered via gavage for seven consecutive days at doses of 5 mg/kg/d or 10 mg/kg/d, followed by animal euthanasia (8 days) and liver and kidney collection for subsequent histological analyses. A one-way ANOVA test was performed, and differences were considered statistically significant at *p* < 0.05. * indicates a difference between treatments and vehicles.

**Figure 4:**
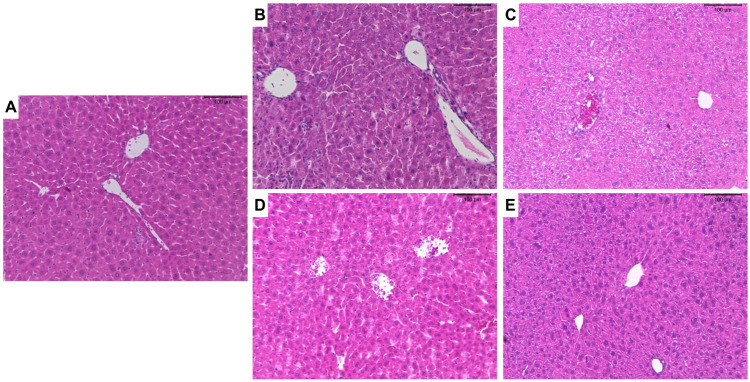
Photomicrographs of the livers of male C57Bl/6 mice after treatment with ZnO nanocrystals or ZnO:9Ag or ZnO:11Ag nanocomposites: group treated with PBS (**A**); groups treated with ZnO9Ag, 5 mg/kg/d (**B**) and 10 mg/kg/d (**C**); groups treated with ZnO11:Ag, 5 mg/kg/d (**D**) and 10 mg/kg/d (**E**). Treatments were administered via gavage for seven consecutive days at doses of 5 mg/kg/d or 10 mg/kg/d, followed by animal euthanasia (8 days) and liver and kidney collection for subsequent histological analyses.

Given that the weight gain in animals treated with 10 mg/kg/d ZnO:9Ag or ZnO:11Ag was reduced, we evaluated whether these nanomaterials were able to induce liver or/and kidney damage. Treatment with 10 mg/kg/d ZnO:9Ag was able to increase the frequency of vesiculation in hepatocytes (++), random inflammatory infiltrate (++) and hyperemia (++) in the organ (Table 3). For treatment with ZnO:11Ag, there was an increase in the frequency of these findings only at a dose of 10 mg/kg/d (Table 3). Through the liver pathological changes index (Figure 3A), it is possible to observe that both ZnO:9Ag (5 mg/kg/d, *p* = 0.0046; 10 mg/kg/d, *p* = 0.0094) and ZnO:11Ag (5 mg/kg/d, *p* = 0.0595; 10 mg/kg/d, *p* = 0.0035) induced mild-to-moderate reversible liver damage (stage II) when compared to the vehicle-exposed group (11–20) (Figure 3A and Figure 4). 

**Figure 5:**
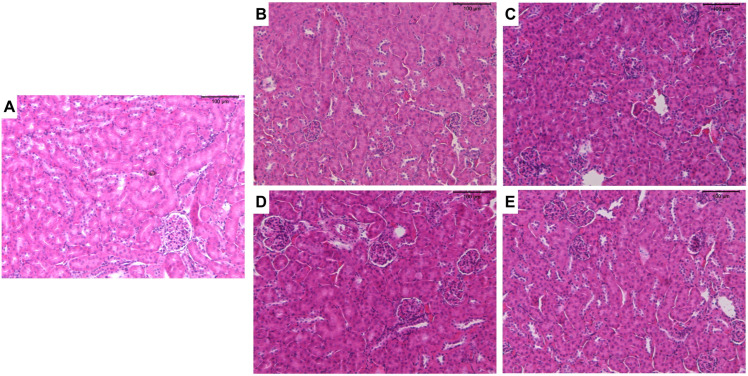
Photomicrographs of the kidneys of male C57Bl/6 mice after treatment with ZnO nanocrystals or ZnO:9Ag or ZnO:11Ag nanocomposites: group treated with PBS (**A**); groups treated with ZnO9Ag, 5 mg/kg/d (**B**) and 10 mg/kg/d (**C**); groups treated with ZnO11:Ag, 5 mg/kg/d (**D**) and 10 mg/kg/d (**E**). Treatments were administered via gavage for seven consecutive days at doses of 5 mg/kg/d or 10 mg/kg/d, followed by animal euthanasia (8 days) and liver and kidney collection for subsequent histological analyses.

With regard to renal tissue, tubules and glomeruli of the cortical region were evaluated. Treatments with ZnO:9Ag or ZnO:11Ag induced vesiculation of renal tubular cells (+) and interstitial hemorrhage (+) (Figure 5), although with low frequency (Table 4). Among the parameters evaluated in the glomeruli, only inflammatory infiltrate (+) and glomerular hemorrhage (+) were reported with low frequency in treatments, regardless of the dose of nanomaterials (Table 4). Finally, through renal HAI (Figure 3B), both nanomaterials, regardless of dose, were able to induce reversible renal damage (Stage II), from mild-to-moderate tissue (11–20), when compared to the vehicle group (ZnO:9Ag: 5 mg/kg/d, *p* = 0.0015 and 10 mg/kg/d, *p* = 0.0023; ZnO:11Ag: 5 mg/kg/d, *p* = 0.0109 and 10 mg/d kg/d, *p* = 0.0004) (Figure 3B). 

Liver and kidney function were also evaluated by measuring alanine transaminase (ALT) and creatinine in the serum of animals treated for seven days and euthanized 8 days after the start of treatment (Table 5). Only ZnO:9Ag treatment showed a tendency to increase ALT levels in serum at the highest doses (10 mg/kg/d *p* = 0.0518). ZnO:11Ag did not induce a significant change in ALT (5 mg/kg/d, p = 0.4777; 10 mg/kg/d, *p* = 0.4051) in the serum of the animals. For creatinine, a marker of nephrotoxicity, both nanocomposites caused a reduction in its levels in the serum of animals treated for 7 days, regardless of the doses used (ZnO:9Ag: 5 mg/kg/d, *p* = 0.0003; 10 mg/kg/d, *p* = 0.0092; ZnO:11Ag: 5 mg/kg/d, *p* = 0.0351; 10 mg/kg/d, *p* = 0.0012).

To evaluate a later period related to the impact of treatment on biochemical parameters, and whether the 5 mg/kg doses would maintain the normal pattern of markers, C57Bl/6 mice were treated for seven consecutive days with ZnO:9Ag and ZnO:11Ag and euthanized 20 days after the start of treatment (Table 5). Interestingly, ALT (ZnO:9Ag: 5 mg/kg/d, *p* = 0.7550; ZnO:11Ag: 5 mg/kg/d, *p* = 0.5656) and creatinine (ZnO:9Ag: 5 mg/kg/d, *p* = 0.6390; ZnO:11Ag: 5 mg/kg/d, *p* = 0.2246) remained at normal values when compared to the control group treated with PBS.

## 4. Discussion

Considering that the association of AgO with ZnO NPs increases the efficiency of their applications, we produced nanocomposites with different concentrations of AgO. Next, we aimed to evaluate whether the amount of Ag in ZnO NPs would interfere with the toxicity of nanomaterials. Accordingly, in vitro and in vivo analyses were performed. It was observed that the in vitro toxicity was dependent on the Ag concentration, with larger amounts being more toxic to immune and nonimmune cells. Further, treatment in invertebrates and mice also demonstrated the same pattern as observed in vitro, with nanocomposites having higher AgO inducing greater toxicity in the animals at higher exposure concentrations. However, it was possible to establish a safe dose for exposure to the nanomaterials based on studies in *G. mellonella* and C57Bl/6 mice.

ZnO NPs decreased the viability of several cell types in vitro, such as HepG2 cells [14], HUVECs [15], THP-1 macrophages [16], peritoneal macrophages [17], Chinese hamster lung fibroblasts (V79 cells) [18], keratinocytes (HaCat) and L929 fibroblasts [11]. In our study, in vitro treatment with ZnO nanocrystals for 24 h resulted in one of the highest CC50 values found for both VERO and RAW 264.7 cells. The association with 49% AgO (ZnO:5Ag) was not able to increase the toxicity of this nanomaterial, as demonstrated by the close CC50 value to the pure nanocrystal (ZnO). However, when higher concentrations of AgO, such as 65% (ZnO:9Ag) and 68% (ZnO:11Ag), were associated, there was a significant decrease in CC50 in both strains. This decrease may indicate that the toxicity is dependent on the AgO concentration, and concentrations higher than 49% AgO NPs may be able to increase the toxicity of ZnO NPs.

Very few studies have evaluated the impact of the association between ZnO NPs and varying concentrations of AgO NPs on cell lines in vitro [11,15]. In comparative studies, for example, it has been reported that Ag NPs were less toxic than ZnO NPs at the same concentrations as HepG2 cells [19]. However, by combining these two metals, it is possible to have a synergic effect. For both Ag and ZnO NPs, some mechanisms are related to cell damage: ion dissolution (Ag^+^ or Zn^2+^), direct contact with the cells, induction of cellular stress processes and mitochondrial dysfunction (oxidative and nitrosative stress), and genotoxicity [19,20,21,22,23,24,25]. Thus, it is believed that the combination of oxides in the ZnO:9Ag or ZnO:11Ag nanocomposites contributes to the cytotoxicity mechanisms in both VERO and RAW 264.7 cells, and hence the higher cytotoxicity when compared to the pure nanocrystal.

The same pattern as observed in vitro was seen in vivo, with animals treated by gavage for seven consecutive days with the highest doses of the nanocomposites ZnO:9Ag or ZnO:11Ag at 10 mg/kg/day demonstrating reduced weight gain. Indeed, one study demonstrated that oral administration of Ag NPs for 7 days at doses of 5, 10, 15 and 20 mg/kg/d was able to reduce weight gain in mice [26]. Moreover, they demonstrated that this type of nanomaterial was related to damage in microvilli, as well as in intestinal glands [26]. Thus, the authors suggest that the loss of microvilli reduces the absorptive capacity of the intestinal epithelium and, consequently, causes weight loss. These results corroborate those found in our study, and the toxicity of AgZnO-AgO nanocomposites is dependent on the concentration of AgO [26]. However, when we evaluated the impact of the 5 mg/kg/d doses for a later period (20 days after the start of treatment), it was shown that the treatment did not negatively impact the weight gain of the animals, especially for the groups treated with ZnO:9Ag and ZnO:11Ag.

Due to these results, we chose to follow biochemical evaluations only in animals treated with ZnO:9Ag or ZnO:11Ag. The biodistribution of ZnO or Ag NPs is well known, with factors such as treatment doses, interaction with body fluids, proteins and cells, NP size, charge, zeta potential and route of administration directly influencing their organ distribution, excretion and toxicity [27,28]. In the case of the oral route, ZnO NPs are absorbed, distributed through the bloodstream and deposited in various organs, especially the liver and kidney [29,30,31]. For Ag NPs, the liver and kidney are also target organs for deposition [32,33,34]. Due to the deposition location for both metals that make up our nanocomposites, these two organs were targeted in our study for hepatoxicity and nephrotoxicity analysis.

Although both treatments, regardless of the dose of ZnO:9Ag or ZnO:11Ag, cause microscopic liver and kidney changes, the lesions are considered reversible and are mild to moderate. The increase in vesiculation of hepatocytes and cells in the renal tubules of the cortex, as well as an increase in random inflammatory infiltrate, hepatic hyperemia and renal hemorrhage, may be a consequence of the sum of the effects of the basic components of the nanocomposites. Consequently, liver and kidney toxicity is also dependent on the insertion of AgO into nanocomposites. 

The nanocomposites showed a low impact on biochemical parameters. Only the ZnO:9Ag nanocomposite was able to induce a tendency to increase ALT at the highest doses and 8 days after the beginning of treatment. In the case of creatinine, in contrast, both ZnO:9Ag and ZnO:11Ag were able to reduce this parameter during the same time interval. This parameter returned to normal when a later period was evaluated (20 days). In this case, different types of ZnO and Ag NPs have already been reported to induce hepatic and renal morphological changes, in addition to altering the same biochemical parameters observed in our study [35,36,37,38,39]. However, the changes reported here are less intense than those reported in the literature. This difference may have occurred due to the low doses of nanocomposites used in our work, the short treatment time, and the production method of the nanocomposites. In addition to these parameters, the route of administration and physiochemical characteristics (size, electrical charge and so on) of the nanomaterials may also influence these results [30,40,41,42].

For ZnO nanoparticles having different synthesis methods and characteristics, size is a parameter that does not interfere with and impact intestinal absorption [3]. However, small ZnO nanoparticles, such as 20 nm, resulted in greater toxicity in an in vitro model when compared to larger sizes, such as 70 nm [43]. Thus, the greater cell toxicity in RAW 264.7 and VERO strains, the reduction in weight of mice and alterations in ALT and creatinine levels related to the ZnO:9Ag and ZnO:11Ag nanocomposites (10 mg/kg) in our work may be associated with the size of these nanomaterials. The higher the doses used, the greater the toxicity in in vitro and murine models [44] (Fujihara; Nishimoto, 2023). Therefore, by reducing the daily dose to 5 mg/kg/d, the nanomaterials proved to be safer.

## 5. Conclusions

Although low, the nanocomposites toxicity was dependent upon the concentration of AgO used in association with ZnO NPs, both in vitro and in vivo. Based upon the results of this study, although soon after the end of treatment there are changes in the weight and biochemical parameters of animals (1 day after the end of treatment), these changes return to normal later (13 days after the end of treatment). Thus, the dose of 5 mg/kg/d for all nanomaterials can be considered safe and of low toxicity. Further, given the screening of the models used, it was possible to establish a safe dose for use in mice.

## Figures and Tables

**Table 1:** Characterization of ZnO, ZnO:5Ag, ZnO:9Ag and ZnO:11Ag nanomaterials using X-ray powder diffraction (XRD) and scanning electron microscopy (SEM).

	Composition (XRD)			Size (SEM)
	ZnO	AgO	Ag	
ZnO	100%	-	-	∼260 nm
ZnO:5Ag	51%	49%	5%	∼250 nm
ZnO:9Ag	35%	65%	9%	∼345 nm
ZnO:11Ag	38%	68%	11%	∼290 nm

Legend: ZnO and AgO: weight % considering only ZnO and AgO mass; Ag: weight % by total mass.

**Table 2:** CC50 and selectivity index of nanomaterials in RAW 264.7 and VERO cell lines.

Nanomaterial	Target Cells	CC_50_ (μg/mL)	Selectivity Index
ZnO	RAW 264.7	95.59	0.79
	VERO	120.6	
ZnO:5Ag	RAW 264.7	112	0.96
	VERO	116	
ZnO:9Ag	RAW 264.7	28.40	1.08
	VERO	26.16	
ZnO:11Ag	RAW 264.7	58.84	1.23
	VERO	47.39	

CC_50_: cytotoxic concentration that causes death in 50% of the cells analyzed.

**Table 3:** Histopathological alterations found in the liver of C57Bl/6 mice in non-treated and treated groups.

Hepatic Changes	Stage	Vehicle	Nanomaterials and Doses			
		PBS	ZnO:9Ag	ZnO:11Ag	ZnO:9Ag	ZnO:11Ag
			5 mg/kg/day		10 mg/kg/day	
Swelling	I	+	+	+	+	+
Vesiculation	I	0	+	+	++	++
Cytoplasmic vacuolation	I	0	0	0	0	0
Inflammatory infiltrate	II	+	++	+	++	++
Hyperemia	II	+	++	+	++	++
Necrosis	III	0	0	0	0	0

Legend: 0 = absence; + = low frequency; ++ = frequent; +++ = very frequent; ++++ high frequency; stage I = reversible liver damage; stage II = more severe repairable liver damage, which affects tissue function; stage III = irreparable liver damage. The animals were treated with ZnO:9Ag or ZnO:11Ag in two doses (5 or 10 mg/kg/day for 7 days), and they were euthanized on the 8th day after the start of the experiment. Liver sections were stained with H&E. Alterations were classified as progressive stages (I, II and III) for the deterioration of liver functions.

**Table 4:** Histopathological alterations found in the kidney of C57Bl/6 mice in non-treated and treated groups.

Kidney Changes	Stage	Vehicle	Nanomaterials and Doses			
Tubular		PBS	ZnO:9Ag	ZnO:11Ag	ZnO:9Ag	ZnO:11Ag
			5 mg/kg/d		10 mg/kg/d	
Dilation of the tubular lumen	I	0	0	0	0	0
Cell peeling	I	0	0	0	0	0
Vesiculation	II	0	+	+	+	+
Hyaline degeneration	II	0	0	0	0	0
Presence of protein in the tubular lumen	II	+	+	+	+	+
Inflammatory infiltrate	II	+	+	+	+	+
Bleeding	II	0	+	+	+	+
Necrosis	III	0	0	0	0	0
Glomerular						
Dilation of capillaries	I	0	0	0	0	0
Thickening of the endothelium	I	0	0	0	0	0
Increase in glomerular volume	I	0	0	0	0	0
Bowman’s space increase	II	0	0	0	0	0
Presence of protein in Bowman’s space	II	0	0	0	0	0
Inflammatory infiltrate	II	0	+	+	+	+
Bleeding	II	0	+	+	+	+
Necrosis	III	0	0	0	0	0

Legend: 0 = absence; + = low frequency; ++ = frequent; +++ = very frequent; ++++ high frequency; stage I = reversible kidney damage; stage II = more severe repairable kidney damage, which affects tissue function; stage III = irreparable kidney damage. The animals were treated with ZnO:9Ag or ZnO:11Ag in two doses (5 or 10 mg/kg/day for 7 days), and they were euthanized on the 8th day after the start of the experiment. Kidney sections were stained with H&E. Alterations were classified as progressive stages (I, II and III) for the deterioration of kidney functions.

**Table 5:** Biochemical parameters evaluated after exposure to ZnO:9Ag and ZnO:11Ag nanocomposites in C57Bl/6 mice at 8 and 20 days after treatment initiation.

Nanomaterial	ALT (U/L)	ALT (U/L)	Creatinine (mg/dL)	Creatinine (mg/dL)	ALT (U/L)	Creatinine (mg/dL)
	8 days	8 days	8 days	8 days	20 days	20 days
	5 mg/kg/d	10 mg/kg/d	5 mg/kg/d	10 mg/kg/d	5 mg/kg/d	5 mg/kg/d
ZnO:9Ag	39.93 ± 4.492	46.57 ± 7.506 ^a^	0.2600 ± 0.02309 ^a^	0.2575 ± 0.05852 ^a^	33.50 ± 6.608	0.2500 ± 0.2311
ZnO:11Ag	34.50 ± 3.915	33.65 ± 9.029	0.2820 ± 0.07430 ^a^	0.2720 ± 0.03701 ^a^	37.50 ± 5.196	0.1833 ± 0.03512
PBS	30.15 ± 9.036		0.4033 ± 0.01528		35.00 ± 6.377	0.3300 ± 0.1735

^a^ when there was a difference between the treatment and the PBS control; ALT: alanine transaminase.

## Data Availability

All the data from this work are available in the article itself and on request from the authors.
